# Population impact of a high cardiovascular risk management program delivered by village doctors in rural China: design and rationale of a large, cluster-randomized controlled trial

**DOI:** 10.1186/1471-2458-14-345

**Published:** 2014-04-11

**Authors:** Lijing L Yan, Weigang Fang, Elizabeth Delong, Bruce Neal, Eric D Peterson, Yining Huang, Ningling Sun, Chen Yao, Xian Li, Stephen MacMahon, Yangfeng Wu

**Affiliations:** 1 The George Institute for Global Health at Peking University Health Science Center, Beijing, China; 2 Department of Preventive Medicine, Feinberg School of Medicine, Northwestern University, Chicago, IL, USA; 3 Division of General Internal Medicine, Department of Medicine, Peking Union Medical College Hospital, Beijing, China; 4 Department of Biostatistics, Duke University, Durham, NC, USA; 5 The George Institute for Global Health, University of Sydney, Sydney, Australia; 6 Duke Clinical Research Institute, Duke University School of Medicine, Durham, NC, USA; 7 Department of Neurology, Peking University First Hospital, Beijing, China; 8 Department of Cardiology, Peking University People’s Hospital, Beijing, China; 9 Peking University Clinical Research Institute, Beijing, China; 10 The George Centre for Healthcare Innovation, University of Oxford, Oxford, UK; 11 Department of Epidemiology and Biostatistics, Peking University School of Public Health, Beijing, China; 12 The George Institute for Global Health at Peking University Health Science Center, Level 18, Tower B, Horizon Tower, No.6 Zhichun Road, Beijing 100088, China

**Keywords:** Cardiovascular disease, Prevention, Primary care, Village doctor, Rural health, China, Cluster-randomized trial

## Abstract

**Background:**

The high-risk strategy has been proven effective in preventing cardiovascular disease; however, the population benefits from these interventions remain unknown. This study aims to assess, at the population level, the effects of an evidence-based high cardiovascular risk management program delivered by village doctors in rural China.

**Methods:**

The study will employ a cluster-randomized controlled trial in which a total of 120 villages in five northern provinces of China, will be assigned to either intervention (60 villages) or control (60 villages). Village doctors in intervention villages will be trained to implement a simple evidence-based management program designed to identify, treat and follow-up as many as possible individuals at high-risk of cardiovascular disease in the village. The intervention will also include performance feedback as well as a performance-based incentive payment scheme and will last for 2 years. We will draw two different (independent) random samples, before and after the intervention, 20 men aged ≥ 50 years and 20 women aged ≥60 years from each village in each sample and a total of 9,600 participants from 2 samples to measure the study outcomes at the population level. The primary outcome will be the pre-post difference in mean systolic blood pressure, analyzed with a generalized estimating equations extension of linear regression model to account for cluster effect. Secondary outcomes will include monthly clinic visits, provision of lifestyle advice, use of antihypertensive medications and use of aspirin. Process and economic evaluations will also be conducted.

**Discussion:**

This trial will be the first implementation trial in the world to evaluate the population impact of the high-risk strategy in prevention and control of cardiovascular disease. The results are expected to provide important information (effectiveness, cost-effectiveness, feasibility and acceptability) to guide policy making for rural China as well as other resource-limited countries.

**Trial registration:**

The trial is registered at ClinicalTrials.gov (NCT01259700). Date of initial registration is December 13, 2010.

## Background

Over the last decade, the global burden of non-communicable disease has increased dramatically [1]. Cardiovascular disease is the leading cause of death and disability worldwide [2]. More than 80% of this disease burden is now borne by low and middle income countries highlighting the need for effective interventions that are affordable, applicable and effective in these settings [3].

There are two widely recognized strategies for the prevention of cardiovascular disease – the “high-risk” strategy and the “population” strategy [4]. However, the debate on which strategy is more effective and cost-effective continues up to date [5,6]. The high-risk strategy is the natural approach for medical practitioners and is recommended by most clinical guidelines as the main strategy for prevention [7-9]. These high risk strategies such as blood pressure, cholesterol and glucose lowering treatment, use of aspirin, and lifestyle counseling among high-risk individuals have been proven both effective and cost-effective through large scale randomized controlled trials, particularly for secondary prevention among those with disease [7]. However, current available studies on the population impact of high-risk strategies were only estimates based on simulation models and parameters from previously published observational studies and clinical trials [10]. There is a lack of evidence from well-designed population-based trial to evaluate the population benefits from implementing these high-risk strategies, particularly in resource-poor settings. Population impact measures can be used to estimate the effect of preventive strategies and interventions on health outcomes at the population level [11] and are therefore useful for informing public health decision making [10].

Rural China is under-developed and has more limited healthcare resources compared to urban areas [12,13]. Chinese rural healthcare system is a 3-level network comprising county hospitals, township healthcare centers and village clinics, and is administrated by county bureaus of health. It provides healthcare for about 700 million rural residents of China, half of the country’s population. The lowest level facility is the village clinic which typically serves a population between 300 and 2500 residents. The primary care health services are provided by “village doctors” who are not qualified physicians by Western standard but are healthcare workers with limited medical training, basic equipment and a restricted pharmacopeia [12,14]. However, the size of this primary care workforce is substantial with an estimated 1.13 million across China [15]. Strategies that enable this workforce to deliver appropriate cardiovascular care to those at high risk have the potential to reduce the disease burden in these resource-poor settings.

The aim of the present study is to assess the population impact of a high-risk strategy focusing on identification of patients at high cardiovascular risk and provision of evidence-based preventive therapies by village doctors. This paper describes the design and rationale of this large, cluster randomized trial.

## Methods

### Overall study design

This study is part of the China Rural Health Initiative, a translational research program to evaluate, at the population level, two evidence-based, practical, simplified, low-cost interventions for prevention and control of cardiovascular disease among 120 rural villages from 10 counties in 5 provinces in northern China. The overall design of the China Rural Health Initiative is two parallel cluster-randomized controlled trials conducted in the same 120 villages but having different interventions and outcomes. The Sodium Reduction Study adopts a population-based strategy to reduce salt intake through health education plus marketing a low-sodium salt substitute while the Primary Care Providers Study uses a high-risk strategy to improve primary care of patients with high risk of cardiovascular disease. We randomly assigned the villages into 4 groups (each having 30 villages): no intervention, sodium reduction intervention only, primary care intervention only, and with both interventions. Each study has 60 villages in intervention and 60 villages in control. The protocol of the Sodium Reduction Study has been published previously [16]. We describe the design and rationale of the Primary Care Provider study here.

Village doctors in the intervention villages will implement the intervention program for identifying and managing patients with high cardiovascular risk after being trained and certified by the study (see below for details) while the control villages will receive no intervention from the study and will continue their practices as usual. The duration of intervention is 2 years. The evaluation of the study includes effectiveness, process and economic evaluations.

### Study setting and participating villages

Five provinces and autonomous regions (Hebei, Liaoning, Ningxia, Shanxi and Shaanxi) will be selected from northern China, where cardiovascular mortality is higher [17-19] and their distances to the study coordinating center in Beijing are shorter than those in southern China. We will then purposefully select two counties from each province and 12 townships from each county giving a total of 120 townships. The two counties will be selected to broadly represent the socioeconomic development of the province. The 12 townships will be selected based on their willingness to participate and proximity to county centers. In order to reduce the risk of intervention contamination, only one village from each township will be invited to participate in the study. The village will not be selected at random but rather on the basis of being located near township centers to maximize the geographic distance between participating villages. At each administrative level, agreement to participate in the study will be sought from relevant authorities.

### Randomization

Using a central computerized process, townships will be randomly assigned to either intervention or control group in a 1:1 ratio. Randomization will be stratified by county in an effort to minimize confounding due to county-level factors such as differing levels of economic development.

### Intervention

The intervention program is designed to enable village doctors to not only identify high-risk individuals but also manage as many eligible individuals as possible so that the population health benefit of the intervention can be maximized. To achieve this goal, our intervention program includes the following components:

1. Technical package for high-risk patient management

The technical package is developed to guide village doctors on how to screen, identify, treat, follow up and refer cardiovascular high-risk individuals during their routine services. The four core technical procedures and corresponding algorithms are given below.

1) Easy identification of high cardiovascular risk individuals:

Village doctors will be required to identify high-risk individuals opportunistically by screening all patients who visit the village clinics for any reason. They will also be encouraged to contact patients with existing diseases or potentially at high risk based on their previous knowledge of the patients to maximize screening.

Patients will be defined as at high risk if they meet one or more of the following criteria:

• History of coronary heart disease or stroke, or

• Older age (men ≥50 years and women ≥60 years) with history of diabetes, or

• Older age (men ≥50 years and women ≥60 years) with measured systolic blood pressure (SBP) ≥160 mmHg.

This definition is based on estimated 10-year absolute risks of a major cardiovascular disease event [20,21]. The age cut-off points in the last two criteria are different for men and women in keeping with the evidence of differential cardiovascular risk according to both age and sex.

2) Evidence-based simple treatments:

Once identified as at high risk, the following basic treatments will be initiated accordingly.

3) Monthly follow-up

• Initiate blood pressure lowering medications if systolic blood pressure ≥140 mmHg and not on blood pressure lowing medication, with low-dose diuretics recommended as first-line therapy due to its efficacy, safety, and low cost.

• Initiate low dose aspirin when SBP < 160 mmHg and not contraindicated

• Provide lifestyle advices such as smoking cessation for current smokers, reduction of dietary salt consumption, weight loss, and physical activity, with emphasis on the first two.

• Encourage use of other evidence-based medications such as β-blockers, ACE-inhibitors or angiotensin receptor blockers and statins, if appropriate and affordable for patients.

All identified high-risk patients will be followed-up every month by the village doctor. At each visit, doctors will measure blood pressure, titrate treatment accordingly, provide lifestyle modification advice and monitor acute symptoms or early signs of clinical events.

4) Timely referral

Patients will be referred to hospital if they are experiencing new cardiovascular events, severe acute symptoms, or if their condition becomes too serious for village doctors to manage in a rural community setting.

2. Training of village doctors and use of assisting tools

Village doctors in intervention villages will be trained using a “train the trainer” model. Cardiologists or general physicians will be selected by county-level health bureaus from the regional county hospital to attend a 2-day training course conducted in Beijing. These physicians will then train the village doctors in their respective counties in two one-day structured sessions, once before the intervention and then again approximately one month after initiation of the intervention. At the end of each training session, all participants will be required to pass an examination to become ‘certified’ in the identification and management of cardiovascular high risk.

**Figure 1:**
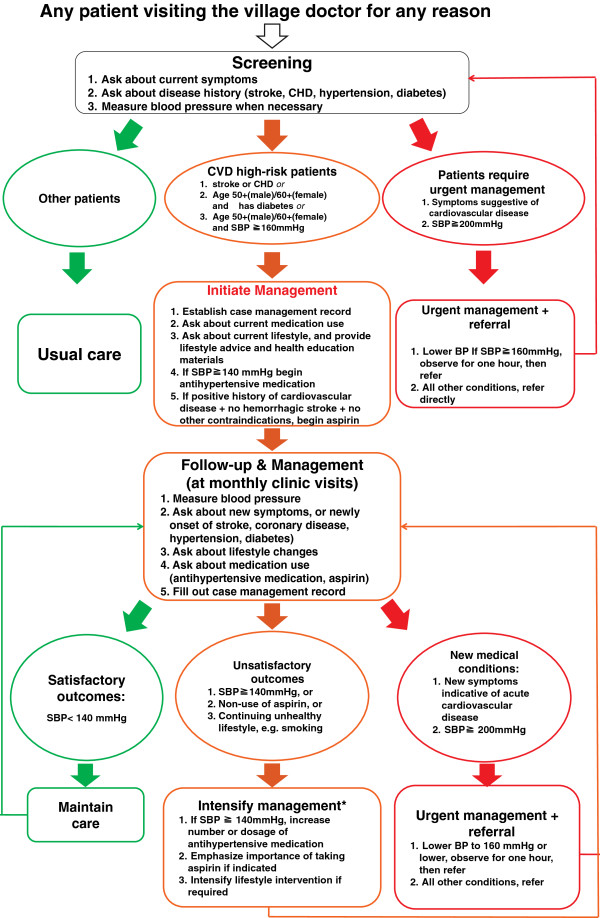
Management algorithm for the identification, management, and follow-up of high-risk patients.

A number of tools will be developed to assist village doctors to implement the standardized management package. A village doctor manual will be developed and provided to village doctors with detailed information on clinical practice procedures, algorithms, medications and standard dosages, etc. to assist the identification, treatment and follow-up of individuals at increased risk of cardiovascular disease. Standard case management forms will also be developed and distributed to village doctors for documenting initial and subsequent patient visits with demographic information, history of disease, medical treatments, lifestyle advice and blood pressure measurements. The summary illustration of the technical procedures and algorithms (Figure 1) will be printed as a 1×1.5 m large colored wall poster and each doctor will be equipped with an electronic sphygmomanometer.

3. Performance feedback

Village doctor performance data from case management records for each patient visit and monthly follow-up will be collected by study staff and entered onto a secure, web-based database from which performance reports will be generated and fed back to village doctors at approximately semi-yearly intervals. The reports will include eight pre-specified key performance indicators (KPIs) relevant to improved cardiovascular risk management. These indicators are number of high risk patients identified, proportion of patients on regular management, proportion of patients with blood pressure under control, proportions of patients given lifestyle advice, low dose diuretics as well as aspirin. The definitions of KPIs are shown in Table 1.

4. Performance-based incentives to providers

**Table 1:** Key performance indicators

**Key performance indicators**	**Definition**	**Duration of measurement**
1. Total number of patients identified	Cumulative number of patients correctly identified by the village doctor	Whole duration of intervention
2. Proportion receiving regular management	Proportion of high-risk patients being regularly managed (with ≥ 5 monthly follow-up records in the past 6 months) by the village doctor in the indicated period	Every 6 months
3. Proportion of patients with blood pressure under control	Proportion of patients with systolic blood pressure under control (with ≥ 5 monthly SBP <140 mm Hg) among those with hypertension	Every 6 months
4. Proportion of patients on life style modification advices	The proportion of patients with case management record indicating the prescription of ≥ 5 months both “salt reduction” and “no smoking”	Every 6 months
5. Proportion of patients on diuretics	Proportion of hypertensive patients with case management records indicating the prescriptions of diuretics ≥ 5 months	Every 6 months
6. Proportion of patients on aspirin	Proportion of high-risk patients with case management records indicating the prescriptions of aspirin ≥ 5 months	Every 6 months

All indicators are based on the case management records (one record per clinical visit) that the village doctors will fill out for all high-risk patients.

In the current healthcare system in China, many of the interventions in our study such as high-risk patient identification and follow-up and lifestyle consultation are not income-generating for village doctors. In addition, we encourage the use of low-cost medications, which usually have a lower profit margin than more expensive ones. To compensate for village doctors’ time and to encourage more preventive services, appropriate amount of KPI-based incentives will be provided by the study to village doctors, in collaboration with local health authorities. These KPIs will also be integrated into village doctors’ annual performance review by their local health authorities.

### Effectiveness evaluation

#### Participants

We will draw two independent, random samples of 40 participants from each participating village for the effectiveness evaluation, one sample for pre- and the other for post-intervention surveys, to collect data for the outcomes measurement. Total number of participants will be 4,800 at each survey. Both pre- and post- intervention surveys will be conducted in the same season from late September to early November to minimize the variation due to seasonal changes. The sampling frame will use the “village roster,” which contains a complete record of all age-eligible adults (men ≥ 50 years and women ≥ 60 years) in the village. Stratified sampling will be used to ensure that each sample contains equal numbers of men and women. We will exclude individuals who have a severe mental health disorder or disabilities that prevent them from effective communication for interview or traveling to the village clinic, or are not able to be exposed with enough time to the intervention (living more than 4 months a year out of village).

#### Baseline and post-intervention survey

The baseline survey will be conducted before the randomization to blind the study staff from the intervention allocation. The second survey will be done after the 2 years of intervention initiation at the same season. Both surveys will be carried out by a study team independent to those involved in the intervention implementation, including village doctors, local project officers, intervention trainers and officials in the county bureau of health. All survey personnel will be trained by the Beijing coordination center in a standardized way and certified through passing a tailor-made examination to become eligible.

Blood pressure, weight, height and heart rate will be measured and recorded by trained study personnel according to standardized protocols. Blood pressure will be measured using an automated electronic sphygmomanometer certified by the European Society of Hypertension International Protocol (Omron Intellisense HEM 7301 IT) [22]. A standardized questionnaire, administered by trained interviewers, will collect information on disease history, lifestyle, medication use, and health care seeking behaviors. Beijing coordination center staff and provincial coordinators will jointly supervise the field work and will monitor the accuracy and completeness of the surveys. Double entry will be used to enter all survey data into Epidata databases by independent professional data entry clerk.

#### Outcomes of effectiveness

The primary outcome for the study will be the difference in mean SBP before and after the intervention. Secondary outcomes will comprise the pre-post differences in indices of clinical care including the proportion of participants (1) receiving regular primary care (defined as monthly visit to village doctor with measurement of blood pressure); (2) receiving lifestyle advice on smoking cessation, reduction in consumption of dietary salt and alcohol, weight control and increasing physical activity; (3) receiving treatment with any evidence-based blood pressure lowing drugs; and (4) receiving treatment with low-dose aspirin.

#### Sample size

The study will be powered on the primary outcome of mean SBP assessed at the individual level. Based on a sample of 4,800 individuals in 120 clusters (townships/villages), an intra-cluster correlation coefficient (ICC) of 0.02 and a standard deviation for SBP of 15 mmHg, the study has 80% power (with two-sided alpha = 0.05) to detect a 1.6 mmHg net difference in pre-post change of SBP and 90% power to detect a 1.9 mmHg net difference in pre-post change of SBP between intervention and control arms.

#### Statistical analysis

The primary analysis will be conducted at the individual level. For the primary outcome of SBP, we will use a generalized estimating equations extension of linear regression model which can account for repeated measurements on the same villages (not individuals), with exchangeable covariance structure, to account for clustering [23,24]. The model will also include a time effect (pre-post) and its interaction with the intervention to test if the pre-post changes are different between intervention and control groups. Point estimates of SBP will be reported with 95% confidence intervals. All analyses will be conducted according to the principle of intention-to-treat. Secondary outcomes will be analyzed using a similar strategy, but logistic regression instead of linear regression will be used for these binary outcomes.

### Process evaluation

A formal process evaluation will be conducted immediately after the post-intervention survey to gain an understanding of the extent to which the intervention is implemented as planned, to evaluate its potential sustainability and to explore enablers and barriers of the intervention. Face-to-face, in-depth interviews with key stakeholders such as local investigators, study coordinators, government officials, project officers, village doctors, and high-risk patients involved in the program will be conducted by independent investigators in selected locations. A standard interview guide will be developed and administered and all interviews will be tape-recorded for later transcription and analysis.

### Economic evaluation

An economic evaluation will be conducted to provide an assessment of the cost-effectiveness of the intervention strategy. Intervention costs will be extracted from financial statements from the project and partner organizations. Patient-level costs including direct and indirect medical costs, productivity loss, and health-related quality of life will be obtained from a cohort of 300 men and women, a random sample of 4,800 baseline survey participants, and the cohort will be followed up every 6 months till the end of the study for collection of personal medical costs data. Cost effectiveness will be assessed, initially in terms of cost per unit change in the primary and secondary outcomes, with additional modeling based on evidence from the literature of disease progression and long-term treatment effects.

### Ethic approval

This study has been reviewed and approved by the Institutional Review Boards of the Peking University Health Science Center in Beijing, China and the Duke University Health System in the US. Cluster-level consent has been obtained through a consultation process involving relevant provincial, county, township, and village authorities. Written informed consent will be sought from all village doctors (before allocation to intervention and control villages) and from all individuals selected for the effectiveness, process or economic evaluations

## Discussion

To the best of our knowledge, this study is the first cluster-randomized trial in the world to assess the population impact of a high-risk strategy in prevention and control of cardiovascular disease. The technical interventions used are all evidence-based and tailored specifically for village doctors. The study design is rigorous and innovative, with large scale, sufficient power, cluster randomization, objective outcomes assessment, independent pre- and post- random samples of target population, blinded evaluation staff, separation of intervention team and evaluation team, sufficient quality control and assurance. The results of the study will provide evidence on the population impact of a high-risk strategy in prevention of cardiovascular disease and help to resolve the dispute around high-risk strategy and population strategy in prevention of cardiovascular disease and other chronic diseases. As a late-stage translational research project, it will also provide important information on feasibility, acceptability, effectiveness, cost-effectiveness as well as possible barriers and drivers for implementation of the low-cost, evidence-based program delivered by village doctors in resource-constrained settings and thus useful to guide policy making for prevention of cardiovascular disease in low-resource countries and settings.

Close attention was paid to feasibility when designing the intervention in this study. In rural areas, the primary healthcare system is substantially under-resourced and village doctors have minimal medical training. A complex strategy for the diagnosis, assessment and management of cardiovascular risk as recommended by most current guidelines [25] (including lab-dependent risk stratification algorithms) is therefore infeasible and unrealistic in rural settings of China. The design of the trial is driven by the need to find a simple, practical and cost-effective solution to improving the management of cardiovascular disease in this resource-limited setting. Village doctors will deliver the intervention program because they are the only health care providers in the villages. In addition, there is good evidence that community healthcare workers can provide effective care with adequate training and technical support [26,27]. Further, the standard of practice provided by such healthcare workers can be improved with interventions that incorporate performance feedback and performance-based incentives [28-30].

The interventions are also designed to be easily incorporated into existing health care policies and payment mechanisms for village doctors. Existing policies apply mainly to the provision of curative services by village doctors and do not extend to preventive care [31]. We therefore designed a performance-based feedback and financial incentive payment, which serves to address this gap. In 2009, China launched a new nationwide health system reform program [32]. Rigorous studies which evaluate the effects of an evidence-based clinical management program delivered by village doctors in such settings are both important and timely. If shown to be effective, the study may encourage Chinese health policy makers to consider an incentive scheme which better caters to non-communicable disease prevention and adopted as a part of the country’s current health system reforms.

To ensure methodological rigor, we incorporated the following design elements: first, we will select only one village from each township to participate in the study. This maximizes the distances between participating villages to reduces the risk of contamination of intervention. Second, to capture the effects of the intervention in improving both the quantity of care (i.e. identification of high risk patients) and the quality of care (i.e. evidence-based practices in management of the high risk patients) and at the same time avoiding cohort effect, we will select two independent random samples -- before and after intervention – in all study villages in preference to a more conventional cluster-randomized trial design of enrolling a fixed number of high-risk patients in each village and following them up, which will not allow us to evaluate the effect of the intervention in improving the quantity of care. Such a design of two independent random samples will also allow us to evaluate the population impact of the intervention, which is the primary aim and innovation of the study.

The evidence generated from the study is expected to have significant implications for policy development in China as a key objective of the current healthcare reforms is to strengthen primary health care [33]. Moreover, the insights gained from this study will have broad applicability to similar, resource-limited settings in other regions of the world where providing effective care at lower cost remains a universal challenge.

## Abbreviations

SBP: Systolic blood pressure; KPI: Key performance indicators.

## Competing interest

The authors declare that they have no competing interests.

## Authors’ contributions

LLY participated in study design and drafted the manuscript. WF, ED, BN, EDP, YH, NS, CY, XL, and SM participated in study design and critical review of the manuscript. YW conceived of the study, participated in its design and critical review of the manuscript. All authors read and approved the final manuscript.

## Pre-publication history

The pre-publication history for this paper can be accessed here:

http://www.biomedcentral.com/1471-2458/14/345/prepub
